# Toll-Like Receptor 9 Agonists for Cancer Therapy

**DOI:** 10.3390/biomedicines2030211

**Published:** 2014-08-04

**Authors:** Davide Melisi, Melissa Frizziero, Anna Tamburrino, Marco Zanotto, Carmine Carbone, Geny Piro, Giampaolo Tortora

**Affiliations:** 1 Digestive Molecular Clinical Oncology Research Unit, Department of Medicine, University of Verona, 10, Piazzale L.A. Scuro, 37134 Verona, Italy; E-Mails: anna.tamburrino@univr.it (A.T.); marco.zanotto88@gmail.com (M.Z.); carmine.carbone@univr.it (C.C.); 2 Medical Oncology, Azienda Ospedaliera Universitaria Integrata, 10, Piazzale L.A. Scuro, 37134 Verona, Italy; E-Mails: melissafriz@libero.it (M.F.); giampaolo.tortora@univr.it (G.T.); 3 Laboratory of Oncology and Molecular Therapy, Department of Medicine, University of Verona, 10, Piazzale L.A. Scuro, 37134 Verona, Italy; E-Mail: genypiro@hotmail.com

**Keywords:** TLR-9, CpG ODN, immune modulatory oligonucleotides, PF-3512676

## Abstract

The immune system has acquired increasing importance as a key player in cancer maintenance and growth. Thus, modulating anti-tumor immune mediators has become an attractive strategy for cancer treatment. Toll-like receptors (TLRs) have gradually emerged as potential targets of newer immunotherapies. TLR-9 is preferentially expressed on endosome membranes of B-cells and plasmacytoid dendritic cells (pDC) and is known for its ability to stimulate specific immune reactions through the activation of inflammation-like innate responses. Several synthetic CpG oligonucleotides (ODNs) have been developed as TLR-9 agonists with the aim of enhancing cancer immune surveillance. In many preclinical models, CpG ODNs were found to suppress tumor growth and proliferation both in monotherapy and in addition to chemotherapies or target therapies. TLR-9 agonists have been also tested in several clinical trials in patients with solid tumors. These agents showed good tolerability and usually met activity endpoints in early phase trials. However, they have not yet been demonstrated to significantly impact survival, neither as single agent treatments, nor in combination with chemotherapies or cancer vaccines. Further investigations in larger prospective studies are required.

## 1. Introduction

The immune system is fully recognized as an important modulator of cancer development and progression. Both innate and adaptive immune cells are involved in such regulation, featuring paradoxical tumor-promoting and tumor-suppressive functions according to the context [1]. The complex interplay of cancer and the immune system can be exploited to re-activate immune surveillance and reverse immune tolerance by cancer immunotherapy. Promising immunotherapies based on recombinant and cellular agents that modulate innate, as well as adaptive immune responses have been described [2]. In particular, current therapies to activate immune effector cells include vaccination with tumor antigens, treatment with cytokines (e.g., interleukin (IL)-2, interferon-α) or enhancement of antigen presentation by the stimulation of different toll-like receptors (TLR) [3].

TLRs belong to the class of pattern-recognition receptors that allow recognition and categorization of pathogens expressing conserved pathogen-associated molecular patterns (PAMPs) in order to activate the most suitable host immune response for the eradication of the infection [4]. Beside exogenous PAMPs, TLRs can recognize endogenous damage-associated molecular patterns [5], molecules released from dead cells or exposed by cells upon stress events. To date, at least 10 members of TLRs family have been identified in humans [6]. The recognition of cognate motifs by TLRs can occur at the plasma membrane [7] or inside the endosomes and phagosomes [4] according to the site of receptor expression. TLR family members 3, 7, 8 and 9 are expressed on the membranes of the endosomes and recognize nucleosides, nucleotides and oligo/poly-nucleotides derived from intracellular viral and bacterial pathogens.

TLR-9 has been commonly considered the specific receptor for unmethylated bacterial or viral CpG motif containing DNA [8,9]. More recently, this view is changing as TLR-9 is considered more in general a receptor for non-self-nucleic acids found in the endosomes, the compartmentalization, rather than the presence of methylated CpG stretches, being the main determinant for signal activation. Barton and colleagues elegantly demonstrated that the intracellular localization was important for the self- and non-self-nucleic acid discrimination [10]. A constitutive expression of TLR-9 has been demonstrated in B-cells and in the type I INF-producing plasmacytoid dendritic cells (pDCs) [11]. Upon activation, additional immune cell types are reported to express TLR-9: human neutrophils [12], monocytes and monocyte-derived cells [13,14] and CD4^+^ T-cells [15]. TLR-9 expression has also been reported in normal human epithelia, including airway epithelium [16] and intestinal epithelium [17], as wells as in human tumor tissues and cells, including breast cancer [18] and cervical squamous cell carcinoma [19].

The rationale that prompted research investigating the possible adoption of TLR-9 agonists in cancer therapy initially relied on their ability to stimulate an inflammatory-like response in order to activate the immune system [20]. Later, it has been demonstrated that TLR-9 agonists can exert antitumor activity by direct action on cancer cells [21,22].

However, from the growing body of literature describing the use of TLR agonists in cancer therapy, it is gradually emerging that they may have conflicting outcomes in distinct cancer settings [23,24]. In this review article, we will report about the role of TLR-9 agonists in cancer therapy with a description of the immune system modulation, as well as of direct tumor cell-related effects shown in preclinical studies and clinical trials.

## 2. Toll-Like Receptors (TLR)-9-Initiated Intracellular Signaling Pathways and Immune Responses

**Figure 1:**
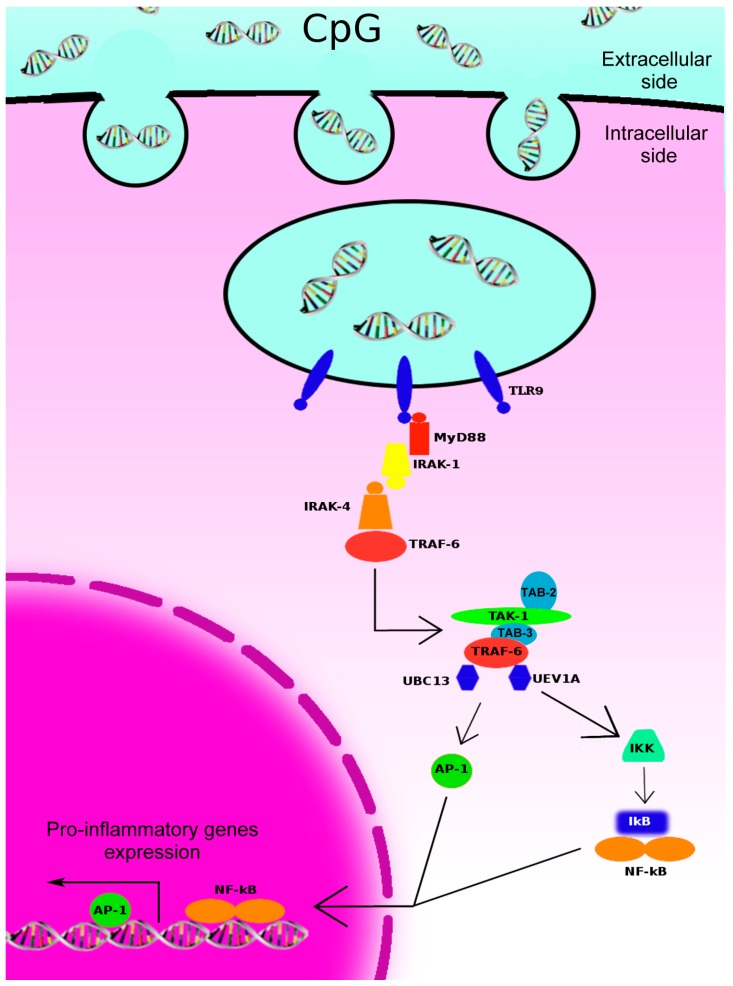
CpG-DNA–TLR-9 cell signaling. CpG oligodeoxynucleotides (ODNs) enter into endosomal vesicles that contain toll-like receptor 9 (TLR-9) through clathrin-coated vesicles. The interaction between CpG-DNA and TLR-9 initiates an intracellular activation signal. The signal starts with the recruitment of myeloid differentiation primary response gene 88 (*MyD88*) to the toll-interleukin-1 receptor (TIR) domain of TLR-9, followed by activation of the IRAK1–TRAF6 complex. Later, TRAF6 recruits TAK1, TAB-2, TAB-3, UBC-13 and UEV-1A. This complex leads to the activation of both the mitogen-activated protein kinase (MAPK: AP-1) and the nuclear factor-κB (NF-κB) kinase inhibitor (IKK), culminating in the up-regulation of transcription factors, including NF-κB and activating protein 1 (AP1). IRAK, IL-1 receptor-activated kinase; TRAF, tumor necrosis factor (TNF)-receptor-associated factor; TAB, TAK1 binding proteins; IκB, inhibitor of kappa B.

The activation of TLR-9 intracellular signaling pathways originates from the toll-interleukin1-resistance (TIR) cytoplasmic domain of TLR-9 after the interaction between the TLR-9 and CpG motif containing DNA internalized in the endosome. This interaction induces the dimerization of TLR-9 that results in allosteric conformation changes in the TIR domains, leading to the recruitment of the adaptor protein myeloid differentiation primary response gene 88 (MyD88) (Figure 1) [25]. MyD88 possesses a TIR domain in the *C*-terminal portion, which is involved in the binding with the TLR-9, and a death domain in the *N*-terminal portion, which is important for the interaction with other downstream proteins [26]. Upon TLR-9 stimulation, MyD88 functions as an adaptor linking TLR-9 with members of the IL-1 receptor-associated kinase (IRAK) family. To date, four different members of the IRAK family have been identified: IRAK-1, IRAK-2, IRAK-M and IRAK-4. All of these proteins consist of two domains, an *N*-terminal death domain, which is responsible for the interaction with MyD88 and other IRAK family members, and a central kinase domain [27]. Different studies revealed that IRAK-4 acts upstream of IRAK-1 and phosphorylates it upon TLR-9-stimulation [28].

IRAK-1 and -4 recruit and activate the RING-domain E3 ubiquitin ligase tumor necrosis factor (TNF)-receptor-associated factor (TRAF) 6 and its accessory factor ubiquitin-conjugating enzyme 13 (UBC13)/ubiquitin E2 variant 1a (UEV1A). This molecular complex is, in turn, responsible for the activation of the serine/threonine kinase in the mitogen-activated protein kinase kinase kinase (MAP3K) family transforming growth factor-β (TGF-β)-activated kinase 1 (TAK1, also called MAP3K7) [29]. TRAF6-mediated K63 ubiquitination of TAK1 leads to the recruitment of the adaptor proteins TAK1 binding proteins 2 (TAB2) or TAB3 through their *C*-terminal Np14 zinc finger (NZF) ubiquitin-binding domain. In this molecular complex, TAK1 autophosphorylates itself at T187 within its activation loop, as well as other sites, including T178, T184 and S192 [30,31,32,33]. Once activated, TAK1 transduces the signal to nuclear factor κB (NF-κB), c-Jun *N*-terminal kinase (JNK) and p38 via phosphorylation of I kappa B kinase (IKK), mitogen-activated protein kinase kinase (MKK) 4/7 and MKK3/6, respectively. Ultimately, NF-κB and other transcription factors downstream of p38 and JNK are activated, resulting in the transcription of genes important for inflammatory and immune responses.

The activation of the TLR signaling plays a pivotal role for the effector functions of both inflammatory and immune cells. During immune response, TLRs have three distinct functions: (1) identifying the type of pathogen; (2) initiating an immediate innate response; and (3) stimulating an adaptive immune response with effector cells appropriate to the specific pathogen [34]. In particular, the activation of TLR-9 signaling in pDCs induces a rapid production of IFN-α [35,36] and tumor necrosis factor α (TNF-α) [37], promoting, in turn, the migration of leucocytes from the bloodstream to the site of infection. IFN-α and TNF-α also initiate the effector functions of innate immunity by inducing the synthesis of antimicrobial peptides and cytokines and activate phagocytosis in the macrophages. The magnitude of the innate response defines the nature of the consequent adaptive immune response [38]. The key element linking the innate to the adaptive immune response is represented by professional antigen presenting cells, such as pDCs. TLR-9 ligands induce the activation of pDCs by CC chemokine receptor 6 (CCR)-6 downregulation and CCR-7 upregulation [39,40]. Thus, pDCs are able to migrate from the peripheral tissue to the draining lymph node [41]. During their migration, activated pDCs produce co-stimulatory molecules, such as cluster of differentiation 80 (CD80) and CD86 [37], molecules of the major histocompatibility complex (MHC) [42] and T helper 1 (Th1) response-promoting cytokines, such as IL-12 and IL-18 [37]. Both co-stimulatory and MHC molecules are necessary for antigen presenting activity of pDCs to naive T-cells, while the activation of the CD4/CD8-immune response requires also the presence of Th1 response-promoting cytokines. All of these steps are triggered by a combination of pathways that can include also the TLR-9 signaling [43].

## 3. TLR-9 Agonists for Cancer Therapy

Over-expression of TLR-9 has been observed in several types of cancer, both in established *in vitro* cells lines and in human cancer samples, including breast cancer, gastric cancer, hepatocellular carcinoma, cervical squamous cell carcinoma, glioma, prostate cancer, colorectal cancer and neuroblastoma [21,22,44]. TLR-9 engagement has been shown to exert both antitumor and tumor promoting effects. In a cell line of hepatocellular carcinoma, TLR-9 activation increases proliferation and inflammation by up-regulating IL-8, IL-1, IL-6, inhibits apoptosis [45] and in esophageal cells stimulates invasion by inducing the expression of *matrix metalloproteinase 2* (*MMP-2*), *MMP-7* and *cyclooxygenase-2* genes [46]. Conversely, evidence has been provided to sustain that TLR-9 activation can cause increased caspase-dependent apoptotic cell death in neuroblastoma cells [21], reduced survival by decreasing Akt activity and antiangiogenic effects by down-regulating vascular endothelial growth factor (VEGF) in colon cancer xenografts [22]. The ability to modulate both immune system response and tumor cells behavior by activating TLR-9 represented, thus, an attractive therapeutic option.

The development of TLR-9 agonists began with the synthesis of three types first generation CpG ODNs, molecules mimicking natural CpG motifs. The first type, A-class, induces a great secretion of IFNα from pDC, but stimulates the B-cell only weakly [47]. The structures of A-class CpG ODN include: three or more consecutive guanines at the 5' and/or 3' ends, which are able to form a very stable, but complex structure, known as G-tetrads, and a central phosphodiester region containing one or more CpG motifs with a palindromic sequence. This features lead the A-class ODN to self-assemble in nanoparticles [48]. The second class of CpG ODN, B-class, has a completely phosphorothioate backbone also in the CpG zone and a structure more linear than the A-class [49]. The members of this class induce both a strong B and NK activation and the secretion of several cytokines, but cause only a weak production of IFNα from pDC. The last class of CpG ODN is the C-class. These molecules are able to induce a strong activation of B-cells, as well as great secretion of IFNα from pDC. These immuno properties seem to be correlated with the unique structure of these ODNs; indeed, they are made up by a stimulatory hexameric CpG motif linked by a T spacer to GC-rich palindromic sequences at the 3' ends [47].

Second-generation CpG ODNs, with advanced nucleic acid chemistry and unique modifications to their sequences and structures, have been developed [50]. Among these TLR-9 agonists, the immune modulatory oligonucleotides (IMOs) are of particular interest because of their potential to induce antitumor immune responses; indeed, they are able to activate directly effector cells, such as NK, and indirectly, through Th1, the cytotoxic T-cells (CTL). Moreover, IMO increases the production of antitumoral cytokines [51] and enhances an antibody-dependent cell-mediated cytotoxicity (ADCC) response through activation of effector cell populations [52,53].

The positive contribution of TLR-9 signaling activation in potentiating antitumor immunity is well established. The tumor microenvironment is characterized by the presence of several immunosuppressive cell types. Among these, the most relevant are the myeloid-derived suppressor cells (MDSCs). High numbers of MDSCs accumulate in and near tumor sites where they inhibit the activity of T and NK cells. Recent studies indicated that the delivery of CpG ODNs directly into the tumor bed could reduce the immunosuppressive activity of monocytic MDSC, by impairing their ability to suppress T-cell function and inducing their differentiation into macrophages with tumoricidal capability [54,55]. Interestingly, a significant decrease of activation of MDSCs has been observed in a B16-OVA syngeneic model of melanoma treated with an oncolytic adenovirus engineered by inserting immunostimulatory CpG islands. This CpG-rich adenovirus demonstrated a more potent antitumor activity when compared with its maternal adenovirus or with the combination of the maternal oncolytic adenovirus plus CpG oligonucleotides [56].

On the other hand, there is a growing body of literature demonstrating that TLR-9 agonist antitumor activity is not only related to their immune response modulation. In the past few years, we studied these aspects and contributed to clarifying several novel mechanisms of IMOs and, more in general, of TLR-9 agonists, demonstrating that their antitumor activity is attributable also to their direct effects on cancer cell behavior. We reported for the first time that TLR-9 physically interacts with epidermal growth factor receptor (EGFR) [52]. The treatment with IMO was able to disrupt this interaction, thus interfering with EGFR-signaling. A combination of EGFR inhibitors with IMO resulted in a synergistic inhibition of Akt and mitogen-activated protein kinase (MAPK) phosphorylation, thus impairing survival and proliferation pathways in colon and pancreatic cancer models [52,57]. IMOs were able to cooperate also with the anti-human epidermal growth factor receptor 2 (HER-2) monoclonal antibody trastuzumab in breast tumors over-expressing HER-2 and resistant to trastuzumab by different mechanisms. Cooperative antitumor activity with cetuximab and trastuzumab was further enhanced by IMO’s ability to boost the ADCC [57,58]. Interestingly, IMO possesses also anti-angiogenic activity, affecting endothelial cells functions and inhibiting VEGF. Furthermore, it potently cooperates with bevacizumab, an antibody devoid of ADCC, in both K-Ras wild-type and K-Ras mutant colorectal cancers [22].

However, some reports showed also opposite effects of TLR-9 agonists in other cancer settings. For instance, the activation of TLR-9 pathway in lung cancer cells enhanced their metastatic potential by indirectly down-regulating tumor suppressive microRNA, such as miR-7 [59]. Similar effects have been observed in human esophageal cancer, where TLR-9 agonists induced the activation of the NF-κB pathway and the expression of MMP-2/-7 [46].

More recently, the TLR-9 pathway was demonstrated also to modulate the expression of DNA repair genes. Whereas TLR-9 agonists reduced the expression of *RAD51*, silent mating type information regulation *2* *homolog (SIRT)-1*, *RAD54B* and *RAD23B* in cancer cells, the same genes were up-regulated in immune cells. These effects sensitized cancer cells to chemotherapy induced DNA damage while preserving immune cells from the same injury [60].

## 4. Clinical Evidences for TLR-9 Agonists Antitumor Activity

The strength of extensive preclinical data [21,22,43,52,54,57,58] supporting the antitumor activity of TLR-9 agonists in many solid tumors prompted us to investigate their tolerability and therapeutic potential in cancer patients, either as single agent treatment, then in combination with standard chemotherapies or cancer vaccines (Table 1).

**Table 1:** Principal clinical trials investigating TLR-9 agonists for cancer treatment.

Agent	Treatment Arms		Study Phase	Cancer Type			No. Patients	Results			References		
PF-3512676	PF-3512676 8 mg *vs*. saline		Phase II randomized	Early stage melanoma			24	In the experimental arm: larger sentinel lymph nodes (SLN), higher SLN leucocytes, higher maturation markers of DC, lower T-reg, increased cytokines			Molenkamp *et al.* [61,62]		
PF-3512676	PF-3512676 0.01–5/10 mg		Phase I	BCC and advanced melanoma			10	Local tumor regression, post-treatment cytokines levels reduction, dense intra- and peri-tumoral lymphocytic infiltrates			Hofmann *et al.* [63]		
PF-3512676	PF-3512676 6 mg		Phase II	Advanced melanoma			20	PR = 10%, CR = 5%, SD = 15% (DCR = 30%)			Pashenkov *et al.* [64]		
PF-3512676	PF-3512676 0.08, 0.12, 0.16, 0.36, 0.54, 0.81 mg/kg		Phase I/II	Metastatic RCC			39	PR = 5%, DCR = 30%			Thompson *et al.* [65]		
PF-3512676	PF-3512676 10 mg *vs*. PF-3512676 40 mg *vs*. PF-3512676 40 mg + DTIC 850 mg/m^2^ *vs*. DTIC 850 mg/m^2^		Phase II randomized	Untreated advanced melanoma			184	Higher ORR (16%) for PF-3512676 40 mg + DTIC 850 mg/m^2^ no differences in mTTP and mOS			Weber *et al.* [66]		
PF-3512676	PF-3512676 0.2 mg/kg + taxane/platinum *vs*. taxane/platinum		Phase II randomized	Untreated advanced NSCLC			117	Higher ORR for PF-3512676 0.2 mg/kg + taxane/platinum (38% *vs.* 19%) Longer mOS PF-3512676 0.2 mg/kg + taxane/platinum (12.3 *vs.* 6.8 ms)			Manegold *et al.* [67]		
PF-3512676	PF-3512676 0.2 mg/kg + CBDCA/TXL *vs*. CBDCA/TXL		Phase III	Untreated advanced NSCLC			828	No significant differences in mOS neither mPFS			Hirsh *et al.* [68]		
PF-3512676	PF-3512676 0.2 mg/kg + CDDP/GEM *vs*. CDDP/GEM		Phase III	Untreated advanced NSCLC			839	No significant differences in mOS neither mPFS			Manegold *et al.* [67]
PF-3512676	PF-3512676 0.2 mg/kg + erlotinib *vs*. erlotinib		Phase II randomized	EGFR mutated advanced pre-treated NSCLC			39	No differences in PFS			Belani *et al.* [69]
IMO-2055 (EMD1201081)	IMO-2055 0.16, 0.32, 0.48 mg/kg + CDDP/5-FU/Cetuximab		Phase Ib	Recurrent/metastatic SCCHN			13	Prematurely stopped for unacceptable toxicity no MTD determined			Machiels *et al.* [70]
IMO-2055 (EMD1201081)	IMO-2055 0.32 mg/kg + cetuximab *vs*. cetuximab		Phase II randomized	Recurrent/metastatic SCCHN never treated with anti-EGFR				Ongoing; recruitment terminated			[71]
IMO-2055 (EMD1201081)	IMO-2055 0.32 mg/kg + FOLFIRI/cetuximab		Phase I	Second-line Kras wt CRC				Ongoing; recruitment terminated			[72]
AS15	MAGE-A3 + AS15 *vs*. MAGE-A3 + AS02b		Phase II randomized	MAGE-A3 positive advanced melanoma			75	Higher ORR (5%) for MAGE-A3 + AS15 Longer 6 ms-PFS (25%) and mOS (33 ms) for MAGE-A3 + AS15			Kruit *et al.* [73]

PF-3512676 (CpG 7909) is a class B CpG ODN that was firstly tested in patients with cutaneous melanoma. This tumor type was shown to be accompanied by a decreased maturation state of DC in the initial tumor-drainage lymph nodes, resulting in the inhibition of the presentation of tumor-associated antigens to specific T-cells [74]. In a small phase II trial, patients with clinical stage I/II melanoma were randomly assigned to preoperative local intradermal administration of either PF-3512676 or saline. In comparison with saline-receiving controls, patients receiving PF-3512676 showed larger sentinel lymph nodes (SLN), higher yields of isolated SLN leucocytes, higher expression levels of maturation markers of DC, lower frequencies of regulatory T-cells and an increased release of a variety of inflammation cytokines [61]. Moreover, increased frequencies of both melanoma-specific CD8^+^ T-cells and NK cells were observed in the SLN from the experimental group [62]. These findings corroborate the potential utility of PF-3512676 as an immunomodulatory adjuvant treatment for early stage melanoma, aiming to minimize the risk of metastatic spread.

The therapeutic potential of PF-3512676 was also investigated in advanced melanoma patients. A phase I trial was performed to evaluate safety, tolerability, anti-tumor activity, serum cytokine levels and cellular immune responses of intralesional administration of PF-3512676 in patients with either basal cell carcinoma (BCC) or cutaneous or subcutaneous metastases of malignant melanoma [63]. Both patients with BCC and metastatic melanoma showed local tumor regression. Serum levels of IL-6, IFN-γ induced protein-10, IL-12 p40 and TNF-α, which were increased post-treatment in most patients. In addition, the majority of lesions of both histological types presented dense intra-tumoral and peri-tumoral lymphocytic infiltrates after PF-3512676 administration.

In a single-arm phase II pilot study, 20 patients with unresectable stage III b/c or stage IV melanoma were treated with weekly subcutaneous injection of PF-3512676 for 24 weeks or until disease progression to assess its tolerability and clinical and immunologic activity [64]. A partial response was observed in two patients, complete response in one patient and stable disease in three patients. Adverse events were generally of modest severity. Immunological assays showed the induction of an activated phenotype of DC, increased levels of a surrogate marker of type I interferon production and a significant stimulation of NK cells.

Given the well-known role of other immunotherapies, such as cytokines [75] and bevacizumab [76], in the treatment of advanced renal cell carcinoma (RCC), PF-3512676 was tested on 39 patients affected by this disease in a phase I/II single arm trial [65]. PF-3512676 was administered subcutaneously every week with cohorts of patients given 0.08, 0.12, 0.16, 0.36, 0.54 and 0.81 mg/kg up to 24 weeks. Overall, the treatment was well tolerated with limited adverse events. Only one dose-limiting toxicity (DLT) was reported, and the maximum tolerated dose (MTD) was not achieved. Twelve patients experienced tumor size reduction and two patients had partial response according to RECIST criteria. PF-3512676 as a single agent generally showed a good safety profile, with only transient local reactions, such as swelling at the injection site and mild flu-like symptoms.

A phase II randomized trial explored the activity of two different doses of PF-3512676 as a single agent treatment or in combination with dacarbazine (DTIC) in 184 patients with unresectable stage IIIb/c or stage IV melanoma [66]. Patients were assigned to four arms of treatment: PF-3512676 10 mg (low dose), PF-3512676 40 mg (high dose), PF-3512676 40 mg plus DTIC 850 mg/m^2^ and DTIC 850 mg/m^2^ as a control. The most frequently reported adverse events were local injection site reactions and systemic flu-like symptoms. The objective response rate (ORR) was greatest in the PF-3512676 40 mg plus DTIC arm (16%), compared with 8% in the DTIC arm, 2% in the PF-3512676 10 mg arm and 0% in the PF-3512676 40 mg arm. Clinical benefit was similar between the PF-3512676 40 mg plus DTIC and DTIC arms. No significant differences in overall survival (OS), nor in median time to progression (mTTP), were observed between the four arms. Thus, the results of this trial did not support the continuation to the phase III portion of the study.

Several preclinical models suggested that TLR-9 agonists can synergize with cytotoxic chemotherapy and anti-EGFR antibodies [6,22,57]. Given the positive results provided by the combination of PF-351267 and paclitaxel in a Lewis murine model of lung carcinoma [47] and the promising efficacy reported in a previous phase II study [67], the addition of PF-3512676 to standard chemotherapy regimens was evaluated as a first line treatment for advanced non-small cell lung cancer (NSCLC) in two phase III trials. The combination of PF-3512676 (0.2 mg/kg subcutaneous Days 8 and 15) and paclitaxel/carboplatin was compared to paclitaxel/carboplatin alone as a control arm on 828 NSCLC patients [68]. No improvement in OS (10 *vs.* 9.8 months, *p* = 0.56), nor in progression free survival (PFS) (4.8 *vs.* 4.7 months, *p* = 0.79), was observed when PF-3512676 was added to standard platinum-based chemotherapy. The second study randomized a total of 839 chemo-naive patients to receive six or fewer cycles of intravenous gemcitabine on Days 1 and 8 and cisplatin on Day 1 alone or in combination with 0.2 mg/kg of subcutaneous PF-3512676 on Day 8 and 15 every three weeks until disease progression or unacceptable toxicity [69]. Median OS and median PFS were similar between the two arms. Moreover, in both of these trials, PF-3512676-receiving patients showed more frequent grade 3–4 hematological adverse events, injection site reactions and flu-like symptoms. Of note, more recently, a phase II randomized trial failed to demonstrate a PFS benefit in favor of the combination of erlotinib and PF-3512676 in EGFR-positive NSCLC patients [77].

Combinations of standard chemotherapy regimens and second-generation TLR-9 agonists, phosphorothioate ODNs, were tested as palliative treatments for patients with advanced stage solid tumors. A phase Ib study investigated the safety of IMO-2055 (EMD1201081) (0.16, 0.32 and 0.48 mg/kg on Days 1, 8, 15 every three weeks) in combination with 5-fluorouracil, cisplatin and cetuximab in patients with recurrent or metastatic squamous cell carcinoma of head and neck (SCCHN) [70]. However, the study was prematurely stopped, because of an unacceptable safety profile without determining the MTD.

In a phase II randomized clinical trial, patients with recurrent or metastatic SCCHN, never treated with anti-EGFR antibodies, were randomly assigned to receive cetuximab alone or in combination with IMO-2055 (weekly subcutaneous injection of 0.32 mg/kg) as a second line treatment. The primary end-point is PFS, and the trial is still ongoing [71].

A dose escalation phase I trial investigated the safety of IMO-2055 in association with a standard regimen containing 5-fluorouracil and irinotecan (FOLFIRI) plus cetuximab in *KRAS* wild-type colon cancer patients who progressed after at least one line of treatment. The study was already terminated, but data have not been released yet [72].

A wide series of early clinical studies explored the potential for different TLR-9 agonists as adjuvants for cancer vaccines. A broad range of compounds consisting of vaccine peptides plus CpG ODNs as adjuvants was tested on small populations of patients with advanced or recurrent melanoma [78,79], renal cell carcinoma and other solid tumors [80,81], showing their ability to successfully stimulate antigen-specific T-cell responses, thus prompting them forward to further phases of experimentation. In a randomized phase II trial, 75 patients with melanoma associated antigen (MAGE)-A3-positive stage III/IV melanoma were treated with MAGE-A3 protein in combination with either AS15, a novel immunostimulant containing a TLR-9 agonist, or AS02b, a different immunostimulant containing a TLR-4 agonist [73]. Four objective responses were observed in the AS15 arm, while only one partial response was observed in the AS02b arm. The six-month PFS and the median OS were 25% and 33 months, respectively, for the AS15 arm and 14% and 19.9%, respectively, for the AS02b arm. The anti-MAGE-A3 cellular response was also more pronounced in the AS15 arm. Thus, the AS15 immunostimulant has been selected as an adjuvant of MAGE-A3 protein for further clinical evaluation.

## 5. Conclusions

TLR-9 recently emerged as a potential therapeutic target for its ability to present non-self-antigens to adaptive immune cells and to stimulate the production of mediators with a direct antitumor activity. The activation of the TLR-9 pathway by using synthetic agonists was thought to be a useful mechanism for the elicitation of the host reaction against cancer. Thus, many TLR-9 agonists, mainly in the form of CpG ODNs, have been developed and explored for the treatment of both hematological and solid tumors.

Solid preclinical evidence had been provided to support multiple mechanisms of action for TLR-9 agonists, either on tumor, endothelial and immune cells, suggesting that this class of agent may play an anti-tumor role, both directly on cancer cells and indirectly on the tumor microenvironment. Indeed, TLR-9 agonists were shown to induce the recruitment of immune effector cells through the activation of pDCs and the consequent copious release of cytokines and to inhibit MDSCs richly present around tumor mass.

Based on this preclinical evidence, a large series of clinical studies has been conducted to verify the relevance of TLR-9 agonists for cancer patients’ treatment. Whereas CpG ODNs as single agent treatments succeeded in demonstrating their ability to potentiate immune response and to induce tumor regression in early phase clinical trials, when tested in combination with conventional chemotherapeutic agents in advanced solid tumor patients, CpG ODNs failed to provide any significant survival advantages over standard regimes, despite increased objective response rates. This lack of survival benefit may be at least partially explained by the advanced stage of disease and the immuno-depressive effect of repeated chemotherapy cycles. Another plausible explanation may lie in the putative differences between human tumor microenvironment and xenograft models, which could make these agents less suitable for stimulating an anti-cancer immune response. Moreover, the most adequate timing for TLR-9 agonist administration is likely to be not yet determined, as an appropriate period between TLR-9 agonists and chemotherapy administration may be necessary to avoid the suppression of anti-cancer immune response by cytotoxic agents.

However, the causes of this failure remain to be better clarified, and ongoing clinical trials are still exploring the combination of TLR-9 agonists and chemotherapy.

Promising results were provided by the combination of TLR-9 agonists and cancer vaccines, although coming from very early and small studies, thus requiring confirmation in larger settings.

Future investigations are expected to be more focused on the combination of TLR-9 agonists with other immunotherapeutic agents, such as vaccines, as combined approaches are more likely to hamper tumor attempts of eluding immune response.

Moreover, TLR-9 agonists will probably find more applications in the treatment of those neoplasms for whom immune components have a more relevant role in their development and maintenance, such as melanoma.

At the present time, solid evidence supporting the use of TLR-9 agonists for the treatment of cancer patients is still lacking. A better understanding of the complex interaction of TLR-9 agonists with the tumor and its microenvironment may have a major value for the clinical development of TLR-9 agonists.
